# Books Reviewed

**Published:** 1872-11

**Authors:** 


### Books Reviewed.

*The Science and Practice of Medicine.* By WILLIAM AITKEN, M. D., Edin., etc. With additions by MEREDITH CLYMER, M D. Third American from the Sixth London Edition. In two Volumes, with Steel Plate, Map, and One Hundred and Eighty Wood Cuts. Philadelphia: Lindsay & Blakiston, 1872. Buffalo: T. Butler & Son.

Fourteen years have elapsed since the first edition of this work was published, and the favorable reception which was given to the former edition will, in a greater degree, be extended to this, the sixth edition, in its greatly enlarged and improved appearance.

The fact that three editions of the work have now been published in this country, evince the fact that it has been read and prized by American as well as by English practitioners.

For eighteen months previous to the publication of the work the author employed himself in a complete revision and correction of its contents.

The nomenclature adopted is that of the College of Physiclans of London, as is also the classification.

The large amount of additional matter which has been incorporated in the work is equivalent to the bulk of a third volume added to the last edition. This increase in amount of matter has been met by a decrease in the size of the type employed, which, however, is clear and distinct, thus preventiug any material enlargement in the bulk of the work. These changes and additions are distributed throughout the whole work, many chapters been considerably enlarged and remodelled, while others are almost entirely rewritten. The topics relative to pathology and morbid anatomy have been to some extent rewritten and greatly expanded ; and the subjects of prevention and treatment of disease are more fully treated of than in any previous edition. To the American editor, Dr. Meredith Clymer, much praise is due for the improvement in the American edition. He has made fragmentary additions to nearly every subject treated, in addition to which some fourteen articles which are presented in this edition are wholly from his pen. Among these we notice articles on Spinal Symptoms in Typhoid Fever, Typho-Malarial Fever, Epidemic Cerebro-Spinal Meningitis, Chronic Pyæmia, Syphilitic Disease of the Liver, Myo-Sclerosic Paralysis, etc. The illustrations and general mechanical execution of the work leave nothing to be desired.

Aitken's practice has long been looked upon as almost indispensable to the practitioner, and the present revised and rewritten edition takes a place, in our mind, in the front rank of treatises upon the Science and Practice of Medicine ; it is, in fact, a complete compendium of the whole subject.

—:o:—

*The Principles and Practice of Surgery.* By FRANK HASTINGS HAMILTON, A. M., M. D., LL. D. New York: William Wood & Co., 1872. Buffalo: T. Butler & Son.

Prof. Hamilton has a world-wide reputation as the author of a Treatise on Fractures and Disloeations, which it is safe to say has no equal in the English language. That as much be said of his present work is, perhaps, unnecessary. The need of such a work as this which he has produced has been for some time felt, and Prof. Hamilton was admirably fitted to perform the task. His work is not one which will be used as a reference on the finer points of surgery and rarer surgical operations ; it is more as a text book for the student and young practitioner. In this respect it takes the place of the more elaborate treatises, which are too cumbersome, too costly, and, on the whole, unsuitable for the student.

The work is divided into two parts—General and Regional Surgery. The nomenclature of the Royal College of Physicians and Surgeons of London has been adopted by the author. He has, however, retained the terms generally used by English and American writers.

The opening chapter on inflammation gives the prominent points with regard to this subject in clear and concise language, and without entering into a discussion of the various theories which have been advanced on this subject, leaves nothing to be desired in its treatment.

In the department of Fractures and Dislocations the author is entirely at home, and has given all that need be included in a work of this kind on the subject.

The portion of the work relating to tetanus is very interesting, and discusses in brief but concise language the different varieties of this affection. We heartily agree with the author in the treatment of Tetanus, believing that inasmuch as the direct lesion causing the affection is not perfectly understood, the treatment should be supporting and anodyne, removing at the same time, if possible, the the cause of the local irritation, instead of any treatment directed to the disease itself.

In the chapter upon gun-shot wounds the author draws largely from his own experience and observation, and gives some valuable hints upon the treatment of this class of injuries.

The work should find a place upon the library shelves of every American surgeon, and may be consulted with the expectatton of finding a clear exposition of the latest views concerning the Principles and Practice of Surgery.

The book may contain some errors; to say that it did not, is to say that the author had accomplished that which no other author ever did. It is, however, as free from faults as any work of the kind of which we have any knowledge.

We take much pleasure, therefore, in recommending the book to our readers, as a treatise upon Surgery, which is entitled to their careful perusal and fullest confidence.

The mechanical execution is every way worthy of the publishers. The illustrations are many of them new, and all are well executed, greatly adding to the ready understanding of the text. We must heartily congratulate both the author and publishers upon the appearance of a work on Surgery, so highly creditable to our American medical literature.

:o:

*The Treatment of Syphilis with Subcutaneous Sublimate Injections.* By Dr. GEORGE LEWIN. Translated by CARL PROEGLER, M. D., and E. H. GALE, M. D. Philadelphia: Lindsay & Blakiston, 1872. Buffalo: T. Butler & Son.

The employment of subcutaneous injections of mercury in the treatment of Syphilis is a treatment which has in the hands of Dr. Lewin met with signal success. The author tells us that he has treated since March, 1865 about fourteen hundred women by this method, and "at the end of July, 1869, there had *not been more than twenty femal patients who had returned to my wards on account of syphilitic relapses*, which were of a very slight character."

This certainly shows an excellent result for the subcutaneous mode of treatment, One of the reasons which the translators give for presenting it to American readers is its rapidity of action and the small amount of mercury used in the treatment, by but one and a half to two and a half grains being employed.

The author, however, is far from thinking that the injection method is the only way of curing syphilis, or that it is applicable in all cases. He says: "I am far from thinking, notwithstanding its remarkable results, that the injection method is the only cure to be adopted, and I earnestly protest against such onesidedness on my part." The subject is one which is every way worthy of careful study on the part of American practitioners, and the book before us presents the subject in all its phases in a manner which will at once be appreciated by the intelligent and observing reader.

:o:

*Epidemic Cerebro-Spinal Menengitis.* With an appendix, on some points on the cause of the Disease as shown by the History of the present Epidemic in the city of New York. By MEREDITH CLYMER, M. D. Philadelphia: Lindsay & Blakiston, 1872. Buffalo: T. Butler & Son.

This little book is a resume of what is known concerning the epidemic of 1864-66, with some valuable suggestions drawn from the present epidemic in New York city. We have often had occasion to express our views concerning the want of knowledge of this disease and its causes, and if Dr. Clymer's observations throw any light on the subject, we shall hail it with gladness. His accurate methods of investigation and his extensive experience have, we hope, lead him to truthful and valuable conclusions.

:o:

*On the Functional Diseases of the Renal, Urinary, and Reproductive Organs, with a general review of Urinary Pathology.* By D. CAMPBELL BLACK, M. D., L. R. C. S., etc. Philadelphia: Lindsay & Blakiston, 1872. Buffalo: T. Butler & Son.

This is an interesting discussion of a subject which the medical practitioner is called upon every day to treat. Dr. Black has given his subject careful and intelligent study, and has taken pains to draw from many sources cases and writings to illustrate his subject. His ideas are some of them new and perhaps a little startling; others are old and long acknowledged, but all are presented in concise terms, with no effort at egotism. To attempt to give a synopsis of the contents of the work beyond that indicated by the heading would, from lack of space, do an excellent little work injustice. We have only to say that it is well written and up to the standard of modern ideas, and that any of our readers need not hesitate to purchase and read it.

:o:

*Carl Both on Small-Pox and Vaccination.* Lee & Shepard, Boston and New York; Trubner & Co., London; Martin Taylor, Buffalo.

This is a strange work, and contains what no other physician would ever think of publishing even, if he entertained such vagaries. He says: "The *predisposition* to small-pox consists in an undue proportion of albuminous matter to the blood-salts, and that as the result, an otherwise inoffensive nervous irritation becomes sufficient to cause the blood to part with this superfluous albumen, which in this case is thrown into the skin, and constitutes that condition which is commonly called smull-pox," He opposes vaccination by unfounded and carelessly made statements, and, on the whole, makes as useless a book as possible ; its only value is as a curiosity, and, for this, is richly worth what it costs—75 cents. It is for sale in Buffalo by Martin Taylor.

———:o:———

*Ovarian Tumors: Their Pathology, Diagnosis and Treatment, Especially by Ovariotomy.* By RANDOLPH PEASLEE, M. D., LL. D., etc. New York: .D Appleton & Co., 1872. Buffalo: Martin Taylor.

The author in his Preface says: "The following work was undertaken from a conviction that a practical treatise, in the English language, upon the subjects of which it treats is greatly needed." At first sight this may appear strange, yet it is the fact that, notwithstanding all that has been written and said upon the subject of Ovariotomy, a complete treatise upon the subject was not to be found in the English language. The various monographs which have been published at different times have been confined to the narration of the experience of their authors in the operation of Ovariotomy and have, with but few exceptions, said nothing concerning the Pathology or Diagnosis of Ovarian Disease.

In the book which is before us we recognize the work of the faithful observer and recorder of facts. Prof. Peaslee has for twenty-five years been engaged in the study of the subject, and from a large experience and careful study, presents a work in every way complete ; nothing that is known upon this subject has been omitted. The views of all authors and the various modes of procedure practiced by them is carefully described, so that it is eminently a practical guide for all who desire direction in this department of Surgery. We notice that the author places our method of enucleation among the established methods of removing Ovarian Tumors, and speaks of it approvingly in several instances. In speaking thus of it as a practical work no one will infer that the descriptions of the anatomy, pathology, classification, and means of diagnosis, are less complete, for certainly no topic in the remotest way connected with Ovariotomy but has received notice. Dr. Peaslee; on Ovarian Tumors, is the standard in our language, and we urge all who propose offering advice to patients suffering from such malady to first carefully study the pages of this author.

